# Supplementary material for: Construction of a Versatile, Programmable RNA-Binding Protein Using Designer PPR Proteins and Its Application for Splicing Control in Mammalian Cells
Source: Cells. 2022 Nov 8;11(22):3529. doi: 10.3390/cells11223529 (PMC9688318; doi:10.3390/cells11223529)
Supplement: Supplementary file 1 [file cells-11-03529-s001.zip › Yagi_etal_Supporting information.pdf]

Supporting information

Construction of A Versatile, Programmable RNA-binding
Protein using Designer PPR Proteins and Its Application for Splicing Control in Mammalian Cells

Yusuke Yagi [1,2,*], Takamasa Teramoto [2], Shuji Kaieda [1,†], Takayoshi Imai [1], Tadamasa Sasaki [1], Maiko Yagi [1],
Nana Maekawa [1] and Takahiro Nakamura [1,2,*]

[1] EditForce, Inc., Fukuoka 819-0395, Japan
[2] Faculty of Agriculture, Kyushu University, Fukuoka 812-8581, Japan
* Correspondence: yusu-yag@editforce.jp (Y.Y.); tnaka@agr.kyushu-u.ac.jp (T.N.)
† Present address: Takara Bio Inc., Kusatsu, Shiga 525-0058, Japan.

Supplemental figures

Figure S1 Sequence logo of PPR2.0
Figure S2 Seamless repeat assembly system for designer PPR gene
Figure S3 Evaluation of the cloning efficiency of designer PPR protein gene
Figure S4 Verification of RBP-ELISA using MS2 RNA binding protein
Figure S5 Detection of Nano-luc fused PPR protein in the lysate
Figure S6 EMSA assay for designer PPR protein of PPR2.0 scaffold
Figure S7 Binding of designer PPR protein with a highly structured RNA
Figure S8 PPR3.0 as a scaffold having improved protein solubility and binding affinity
Figure S9 Design and pre-screening PPR gene for exon 3 skipping of *CHK1* RNA

Supplemental information

Sequence information
Supporting materials and methods

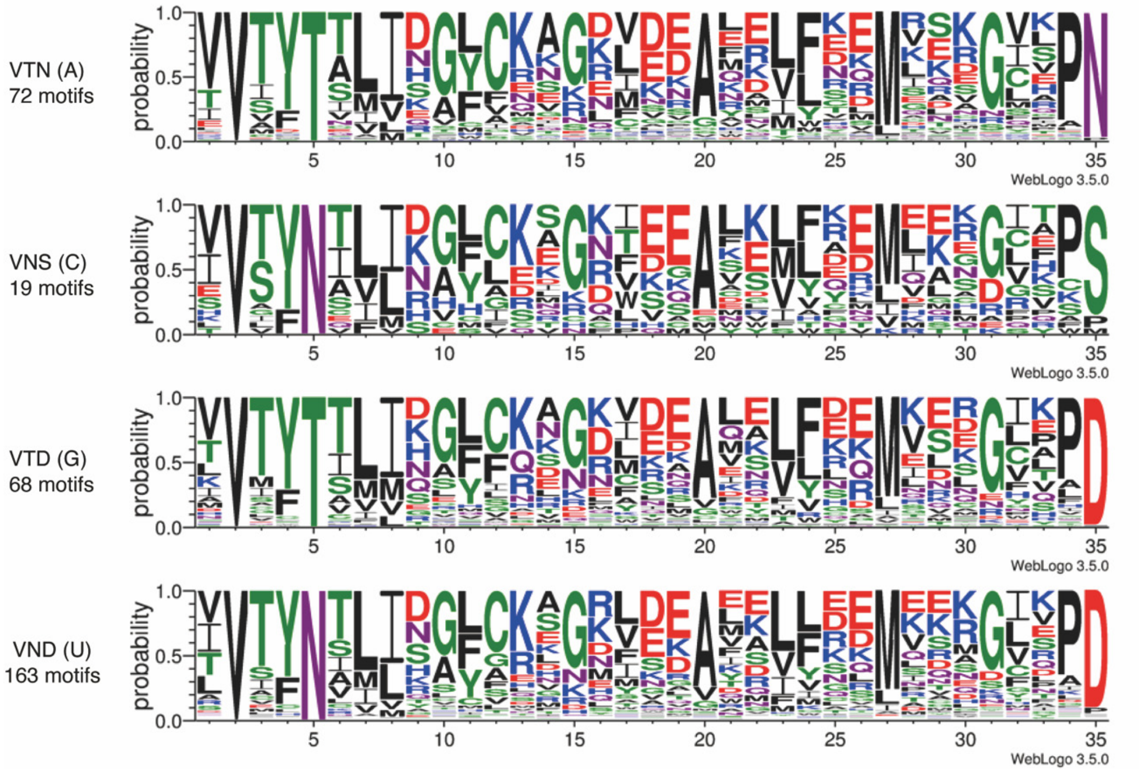

Figure S1 Sequence logo of PPR2.0. Sequence of PPR motifs, which have VTN, VNS, VTD, and VND at 2nd, 5th, and 35th positions, were collected from Arabidopsis PPR sequence information. The sequence logo was created using WEBLogo 3 (http://weblogo.threeplusone.com/create.cgi). Number of PPR motifs used for generation of the sequence logo is shown on the left.

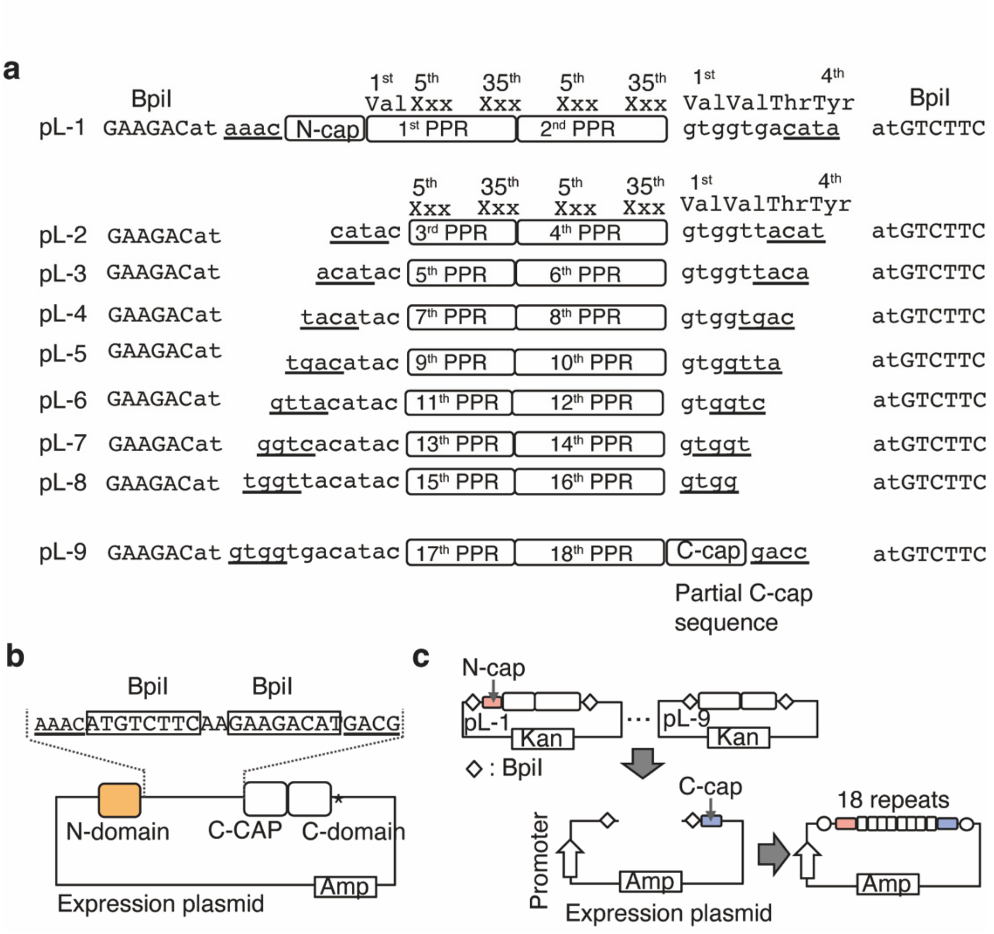

Figure S2 Seamless repeat assembly system for designer PPR gene. (a) Schematic representation of the intermediate plasmids of pL-1 to -9. The pL-1 plasmid encodes N-cap, the 1st and 2nd PPR motifs, and 4 amino acids of the 3rd PPR motif for PPR protein gene containing 18 PPR motifs. The pL-2 plasmid encodes the 5th to 35th amino acids of the 3rd PPR motif, full 35 amino acids of the 4th PPR motif, and 4 amino acids of the 5th PPR motif. In contrast, the pL-9 plasmid contains the sequence for the 17th PPR motif, 18th PPR motif, and partial C-cap sequence. The remaining C-cap sequence is encoded in the expression plasmid. All pL plasmids equip two BpiI sites for the golden gate assembly. **(b)** The expression plasmid is a destination vector of the golden gate assembly, and the plasmid contains the partial C-cap sequence and additional N- and/or C-domain, that is used to construct a PPR fusion gene. The BpiI sites are used for the integration of PPR gene. **(c)** A designer PPR protein gene of 18 PPR repeats containing N- and C-cap structure is assembled, by connecting pL-1 to pL-9 plasmids in order, and integrating this into the expression plasmid using BpiI supporting golden gate assembly. The assembled plasmid can be selected by ampicillin resistance, whereas the intermediate plasmid contains a kanamycin resistant gene.

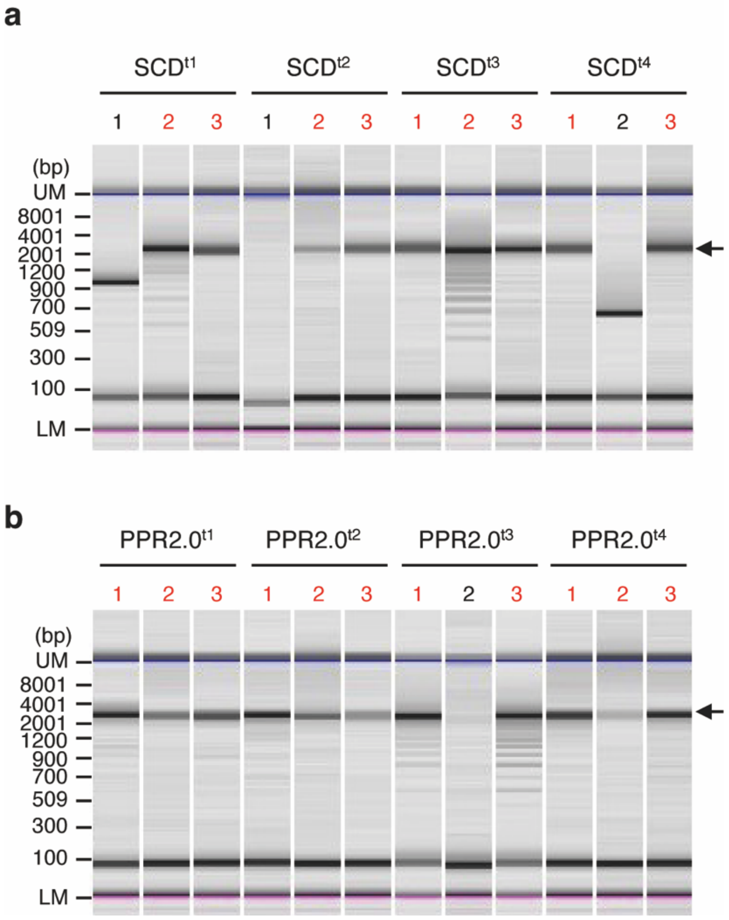

Figure S3 Evaluation of the cloning efficiency of designer PPR protein gene (a) PCR analysis of the assembled designer PPR gene of the SCD scaffold. The SCD^{t1}-SCD^{t4} consist of 18 PPR motifs. Three clones were selected against a single construct. The clone with the correct amplicon size (arrowhead) and sequence is shown in red. **(b)** PCR analysis for the PPR2.0 scaffold (same as that in (a)).

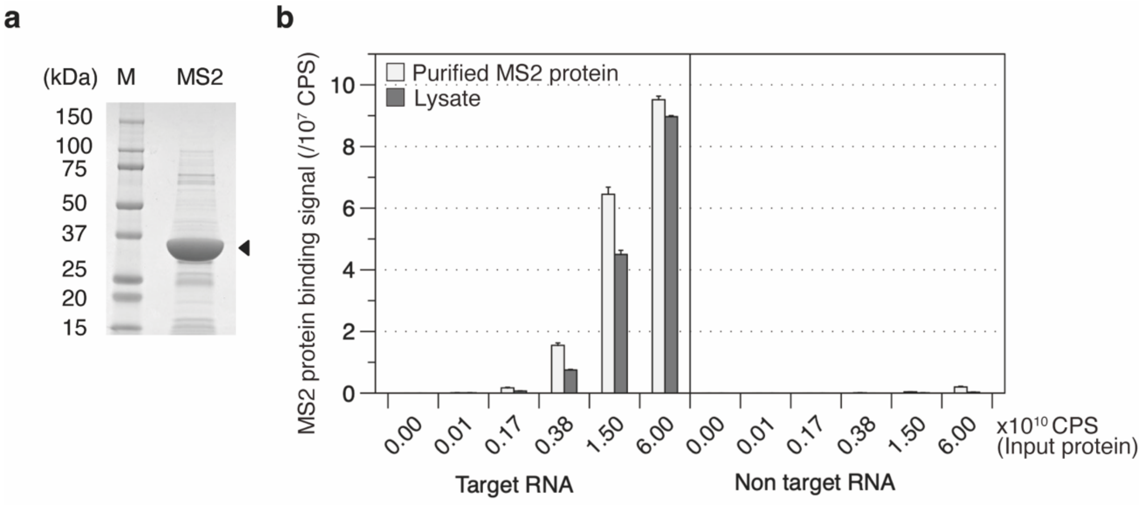

Figure S4 Verification of RBP-ELISA using MS2 RNA binding protein. **(a)** Staining of purified recombinant MS2 protein. The expected size is shown by the arrowhead. **(b)** RBP-ELISA analysis using recombinant MS2 protein with or without purification (N = 3). 0.01, 0.02, 0.09, 0.38, 1.50, and 6.00 × 10^{10} CPS of the purified MS2 protein or the lysate were used. The assay was performed with both target and non-target sequences (listed in Table S4).

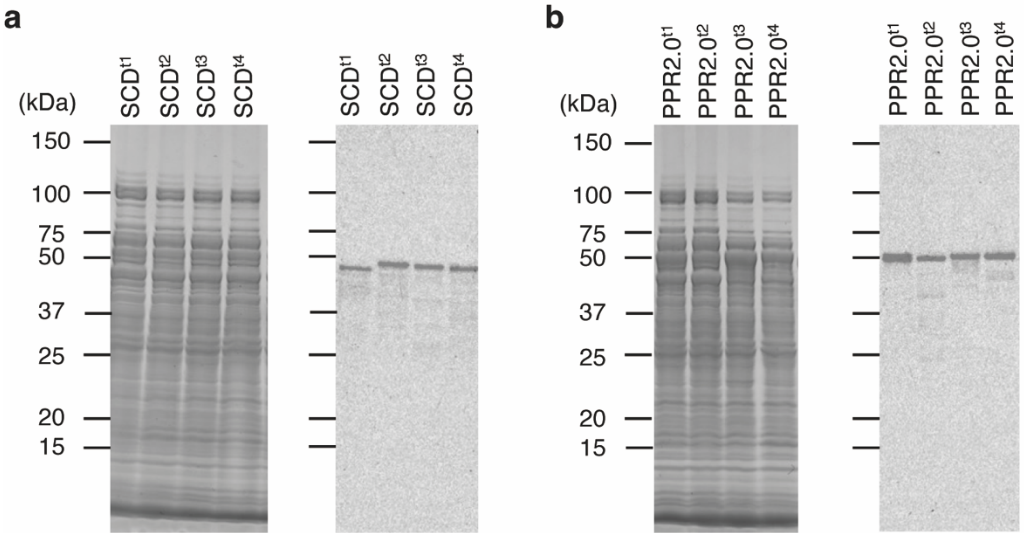

Figure S5 Detection of Nano-luc fused PPR protein in the lysate. The lysate, containing recombinant nano-luc fused PPR protein, was subjected to SDS-PAGE. The CBB stained gel image is shown in the left panel. The Nano-luc protein domain was refolded with 25% isopropanol, and then the luminescence was detected (right panel) to determine the amount of nano-luc fused PPR protein.

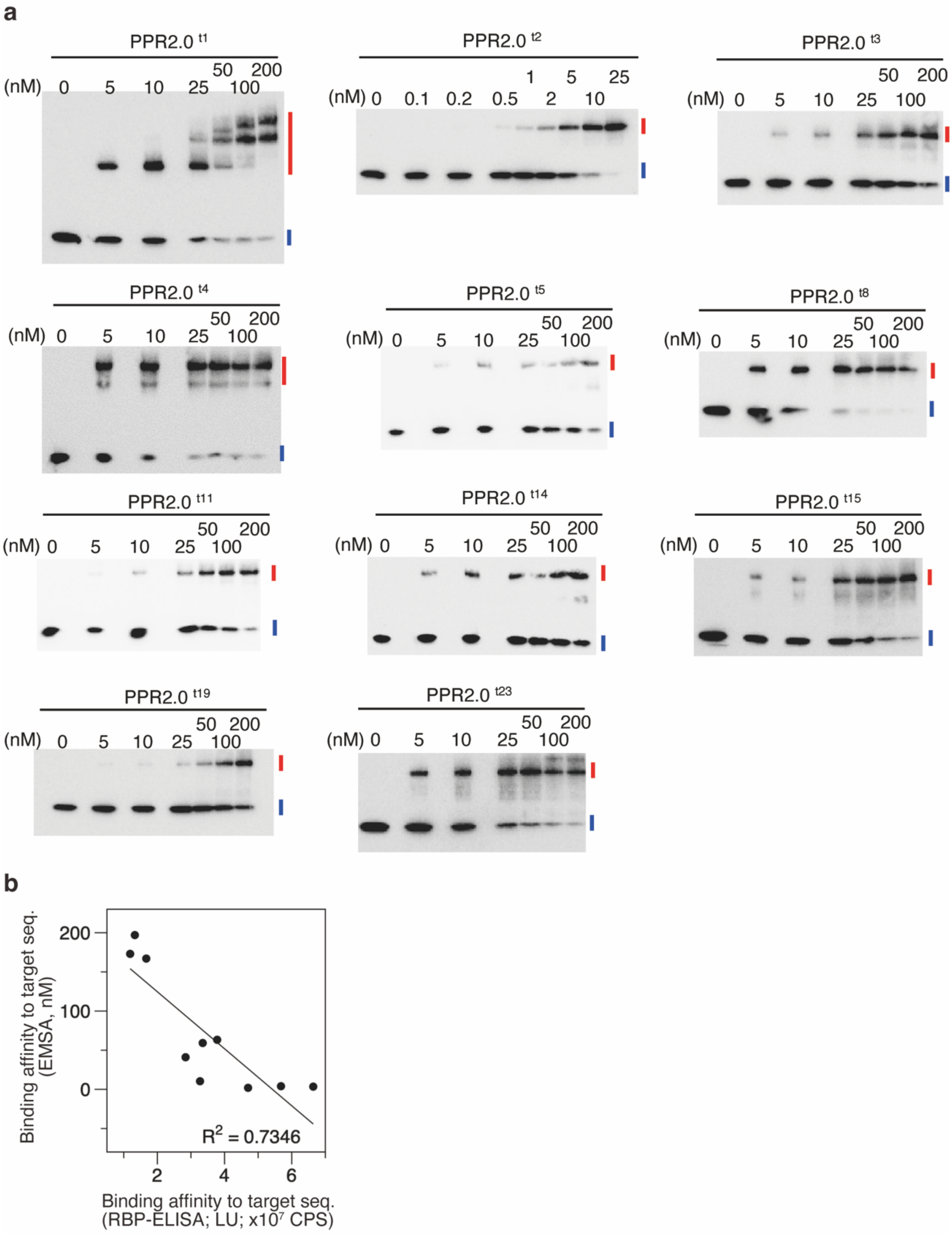

Figure S6 EMSA for designer PPR protein of PPR2.0 scaffold. (a) EMSA was performed to estimate the apparent K_D using various concentrations of the purified recombinant designer PPR protein (0 to 200 nM), and the target RNA. The bound and unbound complexes are highlighted with red and blue lines, respectively. **(b)** The result of EMSA and RBP-ELISA was plotted to estimate the convertibility.

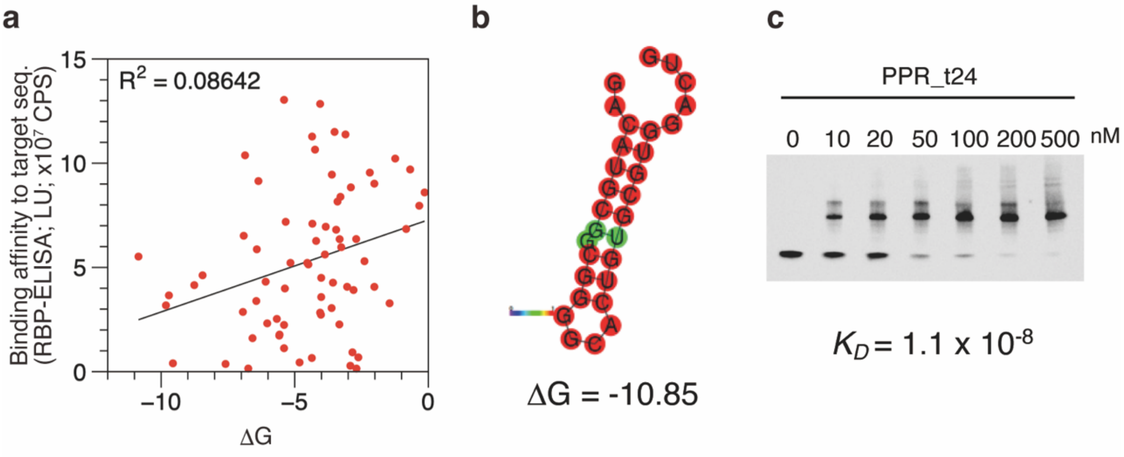

Figure S7 Binding of designer PPR proteins with highly structured RNAs. (**a**) The effect of RNA structure for PPR-RNA interaction was evaluated using 70 designer PPR proteins and plotted, with ΔG of the probe RNA sequence.

(**b**) The predicted secondary structure of t24 RNA probe. The ΔG is also shown. **(b)** EMSA was performed with PPR_t24 protein and t24 RNA probe. Various concentrations of the PPR_t24 protein (0, 10, 20, 50, 100, 200, 500 nM) was used, to estimate the apparent K_D as shown.

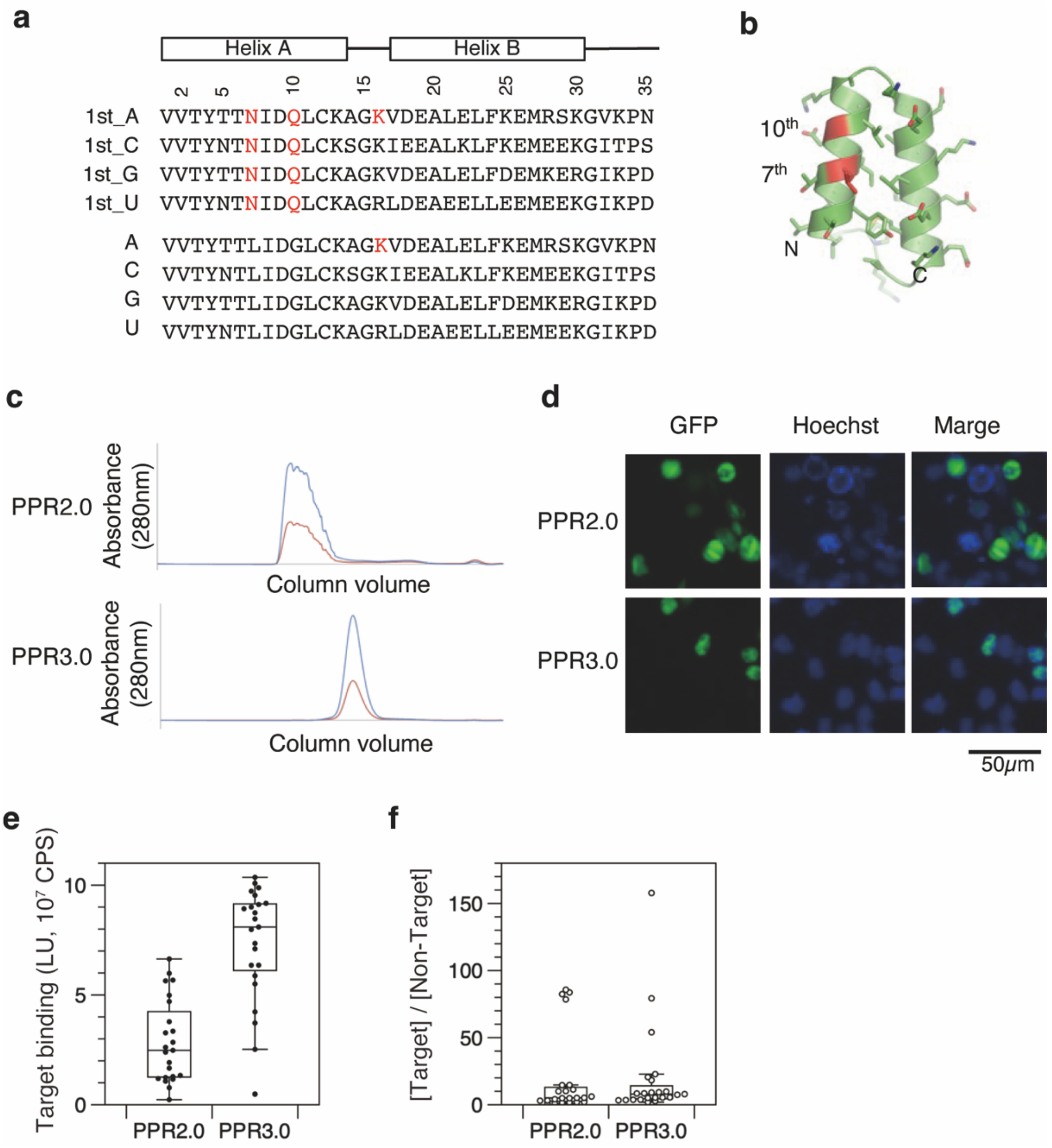

Figure S8 PPR3.0 as a scaffold having improved protein solubility and binding affinity **(a)** Amino acid sequence of PPR3.0 scaffold. The substituted amino acid is highlighted with red, these are the 7th and 10th amino acids of the 1st PPR motif, and 16th amino acids of the PPR scaffolds for adenine recognition. **(b)** The tertiary structure of the PPR motif is shown with the substituted 7th and 10th amino acid. Hydrophilic amino acids were introduced into the 7th and 10th amino acid to increase solubility. **(c)** Gel filtration analysis of designer PPR proteins of PPR2.0 or PPR3.0 (PPR2.0^{t75} and PPR3.0^{t75}; containing 18 PPR motifs) for the evaluation of protein aggregation. Blue and red profiles indicate absorbance at 280 nm and 260 nm, respectively. **(d)** Fluorescent image of the PPR protein in HEK293T cells. The PPR2.0^{t75} and PPR3.0^{t75} are fused with nuclear localization signals and GFP to visualize the cellular localization. **(e,f)** Analysis of RNA binding affinity (e) and

selectivity (f) by RBP-ELISA using 23 designer PPRs with PPR2.0 and PPR3.0. The selectivity was analyzed by comparison of the signal intensity for the target RNA against a non-target RNA.

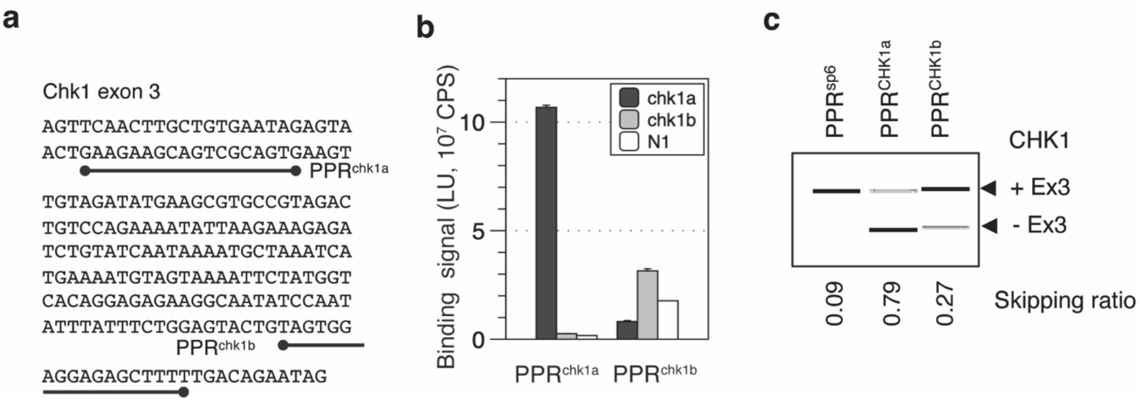

Figure S9 Design and pre-screening PPR gene for exon 3 skipping of CHK1 RNA. a, The partial DNA sequence of exon3 of the *CHK1* gene, and PPR targeted positions. **(b)** RBP-ELISA using PPR^{chk1a} and PPR^{chk1b} **(c)** Pre-evaluation of exon skipping by designer PPR genes of PPR^{chk1a} and PPR^{chk1b} by the transient expression in HEK293T cells. PPR^{sp6} was used as a negative control. The amount of the splicing variant with or without exon 3 (+Ex3, -Ex3) was analyzed using RT-PCR and the skipping ratio was estimated as [-Ex3]/([+Ex3]+[-Ex3]), as shown in Fig. 5.

Supplemental information

Sequence information 1, Amino acid sequence of fusion protein

Amino acid and nucleotide sequence of designer PPRs including CAP motifs are described in Table_S3_PPR list. Sequences of fusion protein that used in each experiments are described in below.

1. Nluc and His-tag fused PPR protein in pETgg_a for RBP-ELISA

Nluc : Red; His-tag : Blue

MAGVFTLEDFVGDWRQTAGYNLDQVLEQGGVSSLFQNLGVSVTPIQRIVLSGENGLKIDIHVIIPYE GLSGDQMGQIEKIFKVVYPVDDHHFKVILHYGTLVIDGVTPNMIDYFGRPYEGIAVFDGKKITVTGT LWNGNKIIDERLINPDGSLLFRVTINGVTGWRLCERILAGGGGSLVPRGSGGGGSSRLH [N-CAP-PPR-C-CAP] SSGSGGSGGGGHHHHHHGR*

2. SBP and His-tag fused PPR protein in pETgg_b for EMSA

SBP : Red; His-tag : Blue

MAGDEKTTGWRGGHVVEGLAGELEQLRARLEHHPQGQREPGGSGGHMGSGGSRLH [N-CAP-PPR-C-CAP] SSGSGGSGGGGHHHHHHGR*

3. His-tag and SUMO-tag fused PPR protein in pETgg_c for gel filtration analysis

His-tag : Red; SUMO-tag : Blue

MGHHHHHHGSLQDSEVNQEAKPEVKPEVKPETHINLKVSDGSSEIFFKIKKTTPLRRLMEAFAKRQG KEMDSLRFLYDGIRIQADQAPEDLDMEDNDIIEAHREQIGG [N-CAP-PPR-C-CAP] SSGMGSGGGR*

4. HA, 3xSV40NLS fused PPR protein for reporter assay and CHK1 exon skipping

HA : Red; 3xSV40NLS

MAGYPYDVPDYAPKKKRKVEDPKKKRKVEDPKKKRKVGGSGGHMGSGGSRLH [N-CAP-PPR-C-CAP] SSGMGSGGGR*

5. EGFP and SV40 NLS fused PPR protein for expression in HEK293T

EGFP : Green; SV40 NLS : Red

MAGVSKGEELFTGVVPILVELDGDVNGHKFSVSGEGEGDATYGKLTLKFICTTGKLPVPWPTLVTTL
TYGVQCFSRYPDHMKQHDFFKSAMPEGYVQERTIFFKDDGNYKTRAEVKFEGDTLVNRIELKGIDFK
EDGNILGHKLEYNYNSHNVYIMADKQKNGIKVNFKIRHNIEDGSVQLADHYQQNTPIGDGPVLLPDN
HYLSTQSALSKDPNEKRDHMVLLEFVTAAGITLGMDELYKPKKKRKVSHGSGGSGGGLH [N-CAP-PPR-C-CAP] SSGMGSGGGR*

Sequence information 2, Amino acid sequence of MS2 protein

We cloned His-tag fused MS2 protein in pET21b vector. Amino acid sequence of the MS2 protein is shown in below.

MAGVFTLEDFVGDWRQTAGYNLDQVLEQGGVSSLFQNLGVSVTPIQRIVLSGENGLKIDIHVIIPYE
GLSGDQMGQIEKIFKVVYPVDDHHFKVILHYGTLVIDGVTPNMIDYFGRPYEGIAVFDGKKITVTGT
LWNGNKIIDERLINPDGSLLFRVTINGVTGWRLCERILAGGGGSLVPRGSGGGGSSRLHASNFTQFV
LVDNGGTGDVTVAPSNFANGVAEWISSNSRSQAYKVTCSVRQSSAQNRKYTIKVEVPKVATQTVGGV
ELPVAAWRSYLNMELTIPIFATNSDCELIVKAMQGLLKDGNPIPSAIAANSGIYLSSGSGGSGGGGH
HHHHHGR*

Supporting materials and methods

RBP-ELISA

1. Instruments
   - Microplate reader (e.g., EnSight, PerkinElmer).
   - Plate shaker (e.g., Maximizer M/BR-420FL, TAITEC)

2. Materials
   - 24 deep well plate (e.g., Microplate 24 Deep-well PP, 10ml/well, Porvair sciences. 360013)
   - Streptavidin-coated 96 well plate (Pierce™, Streptavidin Coated High Binding Capacity White 96-Well Plates with SuperBLock™ Blocking buffer, 15502)

3. Buffers
   - 5× Stock solution (for 1 L)

1 M Tris-HCl (pH 8.0)	100 mL
5 M NaCl	150 mL
10% NP-40	250 mL
D.W.	500 mL
Total	1000 mL

   - 1× Extraction buffer (for 100 samples)

5× Stock solution	6 mL
1 M $MgCl_2$	0.03 mL
50 mg/mL Lysozyme	1.2 mL
100 mM PMSF	0.3 mL
10 mg/mL DNase	0.06 mL
D.W.	22.41 mL
Total	30 mL

   - Binding buffer

5× Stock solution	40 mL
1 M DTT	0.2 mL
BSA	0.2 g

D.W.	159.8 mL
Total	200 mL

- Wash buffer

5× Stock solution	50 mL
1 M DTT	0.25 mL
D.W.	199.75 mL
Total	200 mL

- Blocking buffer

5× Stock solution	4.0 mL
1 M DTT	0.02 mL
BSA	0.2g
D.W.	15.98 mL
Total	20 mL

- Probe solution

20 μM Biotinylated RNA	12.5 μL
5× Stock solution	1 mL
100 mM DTT	50 μL
Nuclease free water	3937.5 μL
Total	5 mL

- Luminescence solution

Dilute 2,500 times Nano-Glo® Luciferase Assay Substrate with Wash buffer

4. Methods

3-1. Culture and protein induction with IPTG

1. Transform bacteria expression plasmids containing Nano LUC fused PPR protein gene into Rosetta™ 2(DE3)
2. Inoculate single colony into 5 mL LB medium in 24 deep-well plate
3. Culture with shaking at 200 rpm, 37 ºC, for 5 to 6 hours.
4. Incubate the deep-well plate at 4 ºC for 30 min.

5. Add 5 µL 100 mM IPTG into a well
6. Culture with shaking at 200 rpm, 15 ºC, for 16 hours.
7. Centrifuge at 3,700 rpm for 15 min at room temperature
8. Remove all medium by pipet or aspirator
9. Pellets can be stored at -20 ºC until use

3-2. Lysate collection

1. Add 300 µL 1× Extraction buffer into the well, and suspend by pipetting
2. Incubate the deep-well plate at -80 ºC for 10 min.
3. Transfer the plate to shaker and incubate at 25 ºC for 30 min with shaking at 200 rpm
4. Centrifuge at 3,700 rpm for 15 min at room temperature
5. Collect the supernatant in a 1.5 mL tube as lysate protein solution

3-3. Measurement of luminescence in the lysate solution.

1. Dilute the collected lysate solution 100,000 times with Wash buffer
2. Mix 40 µL Luminescence solution and 40 µL diluted lysate in the 96 white well plate, and incubate for 5 min at room temperature
3. Measure luminescence using plate reader (PerkinElmer, EnSight)
4. Dilute the lysate solution to 1.5 x 10^8 CPS/µL with Wash buffer

3-4. Preparation of probe solution

1. Prepare 50 nM probe solution (see 2. Buffers)
2. Heat the probe solution at 70 ºC, then immediately cool to 4 ºC for 5 min.

3-5. Binding assay

1. Add 0.2 mL Binding buffer to streptavidin-coated 96 well plate
2. Incubate for 10 min at room temperature
3. Remove buffer by aspiration
4. Add 0.2 mL Binding buffer, then immediately remove the buffer
5. Add 50 µL 50 nM probe solution
6. Seal the plate with parafilm, incubate at 25 ºC for 30 min with shaking at 200 rpm
7. Remove probe solution via aspiration
8. Wash 5 times with 0.2 mL Wash buffer

9. Add 0.2 mL Blocking buffer, then incubate at 25 ºC for 30 min with shaking at 200 rpm
10. Wash 2 times with 0.2 mL Wash buffer, and finally add 0.1 mL Wash buffer
11. Add 0.1 mL Lysate solution and incubate at 25 ºC for 30 min with shaking at 200 rpm.
12. Wash 5 times with 0.2 mL Wash buffer, and add 40μL Wash buffer after final wash
13. Add 40 μL Luminescence solution, and incubate at room temperature for 5 min.
14. Measure luminescence using plate reader (PerkinElmer, EnSight)
